# The Food in Acute Diseases

**Published:** 1888-11

**Authors:** J. Milner Fothergill


### Cullings from Contemporaries.

### THE FOOD IN ACUTE DISEASES.

By J. Milner Fothergill.

A medical education does not yet include any teaching as to what is good in the matter of food in acute illness--in this country at least. So said Sir Wm. Roberts in his address on "Feeding of the Sick" before the British Medical Association in 1885; and his assertion cannot be gainsayed. Nor is it easy to see how long it will be before a less scandalous condition will come into existence. It is understood, both in and out of the profession, that a "slop diet" is the proper thing. But "slops" practically are no more than meat infusions, especially beef-tea, milk and seltzer water, and home-made lemonade. Meat infusions, however, are *not* food, though they have a value of their own. They contain valuable blood-salts and extractives which are stimulant; but they are not "food." They are excellent vehicles for food—as broken biscuit, for instance. Milk should be converted into whey, according to Dr. King Chambers. Lemonade has a certain food-value in proportion to the sugar it contains, and is all the better if made with cream tartar.

What is required by persons acutely sick? That depends upon the temperature. If there be pyrexia present, it is not only useless to give albuminoid matters, as no histogenesis goes on, but there is the positive risk of adding to the waste and excrementitious matters floating in the blood. Soluble carbohydrates and blood salts are what is required. A person may be acutely ill, as in bronchitis, for instance, with but a very trifling rise of temperature, and then milk is not contra-indicated. In such cases milk which has been well-boiled is excellent. The advantages of such boiling are twofold: (1) it destroys disease-germs, and (2) the curd is small and flocculent, and there is no risk of that firm curd which is fraught with so much danger, especially in typhoid fever. It is also very pleasant in the form of junket, or "curds and whey." Some soluble carbohydrate may be added with advantage. But what is a "soluble carbohydrate?" the reader asks impatiently. He ought to have been told in his college course; but it was nobody's duty to tell him, as no examiner would ever ask him such a question. The lecturer on physiology describes to him the act of digestion, and tells how the saliva converts insoluble starch into soluble grape sugar in order that it may pass through the wall of the alimentary canal. But what he has to do when the saliva is scanty and inert is not yet the business of any teacher. He has that to find out for himself—if he can.

The first person who recognised the importance of converting insoluble starch into a soluble maltose was M. Mellin, with his

"non-farinaceous food." He has been followed by a host of imitators, and many excellent predigested foods are on the market. Such foods consist, in the main, of baked flour, or biscuit powder, and malt. They go well with meat infusions, and lend to them a true food-value. With a little salt the whole forms a valuable drink-food, hot or cold. As a food merely the malt extracts possess no advantage over dry maltose. The malt extract is difficult to handle from its viscidity. A dry preparation is easily made into a syrup which will readily pour. Such a malt syrup is most pleasant with an aerated water, and can be iced if desired. A little sharpness can be given to it by a little lemon juice, or lime juice. Or the syrup can be frozen into a nutritive ice. Where the pyrexia is not great it is well to boil milk (one pint) and Mellin's food (a tablespoonful) for an hour. Some of this with an aerated water is most palatable and nutritive. The body wastes in pyrexial disease, and often the lamp of life goes out for want of fuel. If no food be given the sufferer sinks of inanition. If food be given which requires digestion when the digestive ferments are inert, the sufferer dies of starvation just the same. Death from hunger is the hard lot of each. Yes, and myriads perish annually; succumb to this horrible fate, with loving friends around them, anxious to be of service and grudging no expense, while the medical man looks on complacently, and assures them that "everything is being done," unconscious of the abominable falsehood he is uttering. The solemn farce goes on in hundreds of households annually without a scintilla of suspicion being aroused as to the true state of affairs. It is something worse than homicide by misadventure.

What is required is fuel-food in such form as not to require the action of the digestive ferments when these are powerless. The milk sugar of milk; the maltose and dextrine of prepared foods; with water. And some blood salts. In all fevers; in inflammatory conditions; in the acute gastric upsets which occur with delicate children and phthisical patients; in all gastric diseases; and in those conditions of gastric catarrh which follow upon obstruction in the pulmonary circulation, whether due to disease in the heart or lungs; liquid food containing a sufficiency of carbohydrates in soluble form are essential to life. There is no necessity to make the meat infusions too strong. When persons speak of the amount of meat used in the preparatiou of the beef tea given to a certain person who has joined the majority, they do not know what pernicious nonsense they are talking—pernicious because misleading. The food-value of the beef-tea depends upon what is added to it !—*London Hospital Gazette.*

DR. JOS. HOLT, ex-President Louisiana State Board of Health, who made so much fame by the invention and execution of his plan of "maritime sanitation," removed to Portland, Oregon, sometime since. We are pleased to learn that he has been tendered, and has accepted, the chair of obstetrics in the Oregon Medical College.

